# The Availability of Competitive Foods and Beverages to Middle School Students in Appalachian Virginia Before Implementation of the 2014 Smart Snacks in School Standards

**DOI:** 10.5888/pcd12.150051

**Published:** 2015-09-17

**Authors:** Georgianna Mann, Vivica Kraak, Elena Serrano

**Affiliations:** Author Affiliations: Vivica Kraak, Elena Serrano, Department of Human Nutrition, Foods, and Exercise, Virginia Polytechnic Institute and State University, Blacksburg, Virginia.

## Abstract

The study objective was to examine the nutritional quality of competitive foods and beverages (foods and beverages from vending machines and à la carte foods) available to rural middle school students, before implementation of the US Department of Agriculture’s Smart Snacks in School standards in July 2014. In spring 2014, we audited vending machines and à la carte cafeteria foods and beverages in 8 rural Appalachian middle schools in Virginia. Few schools had vending machines. Few à la carte and vending machine foods met Smart Snacks in School standards (36.6%); however, most beverages did (78.2%). The major challenges to meeting standards were fat and sodium content of foods. Most competitive foods (63.4%) did not meet new standards, and rural schools with limited resources will likely require assistance to fully comply.

## Objective

Competitive foods and beverages sold in vending machines, in school stores, at fundraisers, and individually as à la carte cafeteria foods can undermine students’ diet quality and may increase risk of overweight and obesity. However, few studies have assessed competitive foods in rural middle schools (1–3). The purpose of this study was to assess and analyze the availability and nutritional quality of foods and beverages offered to students in vending machines and as à la carte items in 8 rural Virginia Appalachian middle schools before implementation of the US Department of Agriculture’s Smart Snacks in School standards (4). Our hypothesis was that participating schools would have to change 50% or more of foods and beverages to meet the standards.

## Methods

All public middle schools in southwest Virginia in the Appalachian region with 50% or more of students eligible for free or reduced-price lunch under the National School Lunch Program (NSLP) were eligible to participate (5) in this cross-sectional observational study. Of 22 qualifying schools, 11 were randomly selected and contacted. Eight were selected as the study sample. Data were collected in spring 2014, and post-implementation data were collected in 2015.

Trained graduate students conducted audits of school vending machine and à la carte foods and achieved 100% auditor agreement. Auditors used an adapted protocol for vending machines (6) that consisted of each product’s brand name, flavor, variety, price, and package size for each prepackaged item. Only foods available specifically à la carte were audited; NSLP items were excluded because separate nutrition standards apply to them. Nutrition information was obtained directly from the manufacturer or from the product’s nutrition label and was compared with the Smart Snacks in School standards, which align with the most recent Dietary Guidelines for Americans (4). The Smart Snacks in School standards exceed Virginia’s Nutritional Guidelines for Competitive Foods (7). Principals were asked to provide information about existing local school wellness policies to assess the policies included for competitive foods and beverages.

Descriptive statistics on the availability of competitive foods and beverages were computed by competitive food category (à la carte and vending machine) and compared with Smart Snacks standards (4). The percentage of food and beverage items compliant with the new policies was described by standard and by school.

## Results

Four of the 8 participating schools included more than grades 6 through 8: three included kindergarten through eighth grade, and 1 included grades 5 through 8. The average eligibility rate for free or reduced-price lunch was 57.0%; there were no significant differences between participating and nonparticipating schools. More than 93% of students were white. No local school wellness policies on competitive foods and beverages were identified.

Only 4 schools had vending machines. All schools offered water. One school also offered juice in 10-ounce portions, and another offered noncompliant sports drinks. Overall, 36.6% of all à la carte foods and 78.2% of à la carte beverages in each school met all the Smart Snacks in School standards (Table 1). No trend was observed between number of items offered or compliance and eligibility for free and reduced-price lunch. The most popular snack items sold were potato chips, flavored tortilla chips, and other salty snacks. Chips, grain-based desserts, and ice cream often did not meet the standards; however, granola bars and sweet snack mixes did. Common beverages included bottled water (32.4%), carbonated and noncarbonated 100% juice (41.2%), and fruit drinks (23.5%). Some schools offered 5% fruit drinks, which are not permitted under the Smart Snacks in School standards. The most challenging standard to meet was 35% or less calories from fat (62.3%; standard deviation [SD], 19.2%) (Table 2). A high percentage of schools (94.7%; SD, 10.5%) complied with the sugar standard in their foods (≤35% sugar by weight), and most (77.6%; SD, 22.1%) adhered to the saturated fat standard (≤10% saturated fat). Most schools (71.9%; SD, 21.5%) met the 200 calories or less per serving standard.

**Table 1:** Compliance of à la Carte Foods and Beverages With Smart Snacks in Schools Standards, Schools With High Rates of Students Eligible for the Free and Reduced-Price National School Lunch Program, Appalachian Virginia, 2014

School Number	% of Students Eligible for Free and Reduced-Price Lunch	No. of à la Carte Foods Offered^a^	% of Foods Compliant With Standards	No. of à la Carte Beverages Offered^b^	% of Beverages Compliant With Standards
1	51.0	6	16.7	2	50.0
2	51.6	9	55.6	3	66.7
3	56.0	9	22.2	2	100.0
4	56.9	8	25.0	4	75.0
5	58.2	5	40.0	1	100.0
6	59.6	25	36.0	9	66.7
7	59.8	7	42.9	5	80.0
8	63.0	13	53.9	8	87.5
Mean school compliance (95% confidence interval)	—	10.3 (5.8–14.7)	36.5 (26.6–46.5)	4.3 (2.2–6.3)	78.2 (66.2–90.3)

Abbreviation: —, not applicable.

a Foods offered include exempted food items (eg, part skim cheese).

b The same beverage offered in a different size was counted as a separate beverage.

**Table 2:** Schools in Compliance with Smart Snacks in School Standards for à la Carte Foods, by Nutrient Category Standard, Appalachian Virginia, 2014

School Number	% of Students Eligible for Free and Reduced-Price Lunch^a^	Foods Meeting Smart Snacks in School Standards							
		≤200 Calories	≤35% Sugar by Weight	≤35% Calories From Fat	<10% Calories From Saturated Fat)	<0.5g Trans Fat	≤230 mg Sodium	Ingredient Standards	All Standards
1	51.0	50.0	100.0	33.3	100.0	100.0	66.7	83.3	16.7
2	51.6	77.8	100.0	55.6	100.0	100.0	66.7	77.8	55.6
3	56.0	100.0	100.0	44.4	44.4	100.0	100.0	77.8	22.2
4	56.9	37.5	100.0	50.0	62.5	100.0	50.0	100.0	25.0
5	58.2	60.0	100.0	80.0	80.0	100.0	40.0	80.0	40.0
6	59.6	72.0	72.0	80.0	80.0	100.0	60.0	88.0	32.0
7	59.8	85.7	85.7	85.7	100.0	100.0	71.4	85.7	42.9
8	63.0	92.3	100.0	69.2	53.9	100.0	100.0	92.3	53.9
Average compliance by school, % (SD)	—	71.9 (21.5)	94.7 (10.5)	62.3 (19.2)	77.6 (22.1)	100.0	69.3 (21.5)	85.6 (7.7)	36.0 (14.5)
Average compliance by food item, % (SD)	—	74.4	90.2	65.9	75.6	100.0	70.7	86.6	36.6

Abbreviation: SD, standard deviation; —, not applicable.

a Values are percentages unless otherwise noted.

Compliance with individual standards by schools and by food items was similar but not identical. Some schools offered more food items than others (Tables 1 and 2). Most foods (85.6%; SD, 7.7%) met ingredient standards and 36.6% of competitive food items were compliant with all Smart Snacks in Schools standards.

## Discussion

Findings validated the stated hypothesis that at least 50% of items would need to be replaced with reformulated or alternative foods and beverages, because 63.4% of à la carte and vending machine food items did not meet the new standards (8). Fat and sodium restrictions proved to be the most difficult standards to achieve, and items included flavored tortilla chips and ice cream novelties (fat) and chips, cheese crackers, and baked goods (sodium). Food items that failed to meet the caloric restrictions often met the other standards, with the exception of serving size. Most food items met the sugar restriction except ice cream novelties. Flavored tortilla chips failed the ingredient standards. Based on data from the vending machines, only one school would need to change vending offerings based on the new standards, which does not align with our hypothesis.

The number of items offered à la carte did not correlate with the percentage of eligibility for free and reduced-price lunch in schools, in contrast to literature showing lower purchasing power for competitive foods among students who are eligible for free and reduced-price meals (9–11). Each county in the study offered different numbers and types of foods and beverages even within the same region. Item variety offered in comparison to previous research implied that more of the schools in the present study provided ice cream; fewer provided sugar-sweetened choices and similar sweet and salty snack options (12). The primary limitations of this study were the small school sample size and lack of external validity.

Future research should assess how competitive foods contribute to students’ diet quality and how new standards affect NSLP participation. Qualitative studies are needed to elicit feedback about the opportunities, challenges, and resources needed to help rural middle schools implement the Smart Snacks regulations.
