# Erratum, Vol. 12, September 17 Release

**DOI:** 10.5888/pcd12.150051e

**Published:** 2015-10-08

In the article “The Availability of Competitive Foods and Beverages to Middle School Students in Appalachian Virginia Before Implementation of the 2014 Smart Snacks in School Standards,” the author made an error in calculating the order of data presented in Table 2. Changes do not affect study results. Numbers changed in all columns and rows of Table 2. In addition, changes were made to the text.

The following changes marked in bold were made to the Results section:

Only 4 schools had vending machines. All schools offered water. One school also offered juice in 10-ounce portions, and another offered noncompliant sports drinks. Overall, **36.6%** of all à la carte foods and 78.2% of à la carte beverages in each school met all the Smart Snacks in School standards (Table 1). No trend was observed between number of items offered or compliance and eligibility for free and reduced-price lunch. The most popular snack items sold were potato chips, flavored tortilla chips, and other salty snacks. Chips, grain-based desserts, and ice cream often did not meet the standards; however, granola bars and sweet snack mixes did. Common beverages included bottled water (32.4%), carbonated and noncarbonated 100% juice (41.2%), and fruit drinks (23.5%). Some schools offered 5% fruit drinks, which are not permitted under the Smart Snacks in School standards. The most challenging standard to meet was 35% or less calories from fat (**62.3%; standard deviation [SD], 19.2%**) (Table 2). A high percentage of schools (94.7%; SD, 10.5%) complied with the sugar standard in their foods (≤35% sugar by weight), and most (77.6%; SD, 22.1%) adhered to the saturated fat standard (≤10% saturated fat). Most schools (**71.9%; SD, 21.5%**) met the 200 calories or less per serving standard.

Compliance with individual standards by schools and by food items was similar but not identical. Some schools offered more food items than others (Tables 1 and 2). Most foods (**85.6%; SD, 7.7%**) met ingredient standards and **36.6%** of competitive food items were compliant with all Smart Snacks in Schools standards.

The following changes marked in bold were made in the first paragraph of the Discussion section: “Findings validated the stated hypothesis that at least 50% of items would need to be replaced with reformulated or alternative foods and beverages, because **63.4%** of à la carte and vending machine **food** items did not meet the new standards (8).”

The changes were made to our website on September 17, 2015, and appear online at http://www.cdc.gov/pcd/issues/2015/15_0051.htm. We regret any inconvenience or confusion this error may have caused.

